# In Search of State and Trait Emotion Markers in Mobile-Sensed Language: Field Study

**DOI:** 10.2196/31724

**Published:** 2022-02-11

**Authors:** Chiara Carlier, Koen Niemeijer, Merijn Mestdagh, Michael Bauwens, Peter Vanbrabant, Luc Geurts, Toon van Waterschoot, Peter Kuppens

**Affiliations:** 1 Department of Psychology and Educational Sciences Katholieke Universiteit Leuven Leuven Belgium; 2 Department of Smart Organisations University College Leuven-Limburg Heverlee Belgium; 3 Department of Computer Science Katholieke Universiteit Leuven Leuven Belgium; 4 Department of Electrical Engineering Katholieke Universiteit Leuven Leuven Belgium

**Keywords:** depression, emotions, mobile sensing, language, LIWC, openSMILE, speech, writing, mobile phone

## Abstract

**Background:**

Emotions and mood are important for overall well-being. Therefore, the search for continuous, effortless emotion prediction methods is an important field of study. Mobile sensing provides a promising tool and can capture one of the most telling signs of emotion: language.

**Objective:**

The aim of this study is to examine the separate and combined predictive value of mobile-sensed language data sources for detecting both momentary emotional experience as well as global individual differences in emotional traits and depression.

**Methods:**

In a 2-week experience sampling method study, we collected self-reported emotion ratings and voice recordings 10 times a day, continuous keyboard activity, and trait depression severity. We correlated state and trait emotions and depression and language, distinguishing between speech content (spoken words), speech form (voice acoustics), writing content (written words), and writing form (typing dynamics). We also investigated how well these features predicted state and trait emotions using cross-validation to select features and a hold-out set for validation.

**Results:**

Overall, the reported emotions and mobile-sensed language demonstrated weak correlations. The most significant correlations were found between speech content and state emotions and between speech form and state emotions, ranging up to 0.25. Speech content provided the best predictions for state emotions. None of the trait emotion–language correlations remained significant after correction. Among the emotions studied, valence and happiness displayed the most significant correlations and the highest predictive performance.

**Conclusions:**

Although using mobile-sensed language as an emotion marker shows some promise, correlations and predictive *R*^2^ values are low.

## Introduction

### Background

Emotions are crucial to human survival, functioning, and well-being. They alert us to opportunities and challenges in our environment and motivate us to act on them to serve our goals and concerns [1]. As such, how people feel throughout their daily lives is an important determinant of their overall mental well-being [2,3]. On average, feeling higher levels of positive emotions and lower levels of negative emotions is generally considered to reflect better well-being, and mood disorders involve extreme instantiations of this [4]. Aside from average levels, emotions are in constant movement and fluctuation over time [1,3,5]. Small but repeated deviations in moment-to-moment emotion dynamics can accumulate over time into larger deviances in mood and, ultimately, episodes of mood disorders. Therefore, reliable and suitable methods to measure people’s daily life emotions, in terms of both momentary fluctuations and average levels, are much needed to further improve the study of emotion and emotion disorder and help in the detection and prevention of maladaptive emotional functioning. One of the ways in which people convey emotions is language. In this paper, we will examine to what extent language-based data collected through mobile sensing can be instrumental in the prediction of emotions.

### Experience Sampling Method

The current gold standard for researching emotion (dynamics) in daily life is the experience sampling method (ESM). Participants complete a short survey on how they feel multiple times a day, allowing data to be collected during their normal routine [6]. The momentary nature of the assessment helps mitigate memory biases, enhances ecological validity, and allows for within-person patterning and investigating of relationships [7-9].

However valuable, the ESM has some drawbacks. Interrupting daily activities for a survey multiple times a day can be burdensome [10]. Motivation loss may induce untruthful or superficial responses, compromising data quality [11]. Furthermore, thinking about emotions multiple times a day may influence their natural flow [9,12], and social desirability in self-reports is a known problem [9]. These drawbacks could be avoided if it were possible to collect equally informative data without having to rely on the participants’ active involvement.

### Mobile Sensing and Language

One such unobtrusive (passive) data collection method as an alternative to ESM is mobile sensing [13]. Whenever we use or carry our mobile devices, mobile sensors and user logs such as light sensors, accelerometers, and app use logs are registered as traces of our digital behavior [14,15]. Given the pervasiveness of smartphones, this continuous flow of information might enable the automatic and unobtrusive detection of behavioral features such as sleep, social behavior, or even mood disorder episodes to aid in research and clinical practice [14,16-19].

We need emotionally valid data that can be captured by a smartphone to be able to use mobile sensing in the detection of emotion and mood disorders. Language is one of the ways in which people (digitally) express their emotions [20]. Both language and emotions also serve as communication and cooperation tools and mutually influence each other [21]. People explicitly or implicitly convey emotions to their interaction partners through what they say and how they say it [22-26]. Therefore, in this paper, we will examine to what extent language-based data collected through mobile sensing can be instrumental for the prediction of momentary and trait emotion. We make a distinction, on the one hand, between what people communicate (content) and how they communicate it (form) and, in contrast, between speech and writing, resulting in 4 types of language data (Textbox 1).

**Types of language data.**

**Types of language data**

- Speech content: spoken words
- Speech form: voice acoustics (eg, pitch and timbre)
- Writing content: written words
- Writing form: typing dynamics (eg, typing speed and key press duration)

### Previous Related Work

#### Speech Content

Studies on speech and emotional word use have generally focused on positive or negative emotions. Induced positive emotions coincide with more positive and less negative emotions between persons [27,28]. In addition, in natural language snippets, a positive association between trait positive affectivity and positive emotion words was found [29]. Higher trait negative affectivity and higher within-person negative emotions coincided with more negative emotions and more sadness-related words in experimental and natural settings [27-29]. However, a recent study did not find any significant correlations between emotion words and self-reported emotions either within or between persons [30].

Because of these inconsistencies, *The Secret Life of Pronouns* supports the use of nonemotion words to assess emotional tone. In particular, depression and negative emotionality show a small correlation with first-person pronouns [31,32]. A larger variety of studies was conducted with writing, which will be further addressed in the *Writing Content* section.

#### Speech Form

Each voice has a unique sound because of age, gender, and accent. However, psychological features such as attitudes, intentions, and emotions also affect our sound [26]. Johnstone and Scherer [33] discern three types of features: time-related (eg, speech rate and speech duration), intensity-related (eg, speaking intensity and loudness), and features related to the fundamental frequency (F0; eg, F0 floor and F0 range). A fourth type could be timbre-related features (eg, jitter, shimmer, and formants). (Mobile-sensed) voice features have repeatedly been used in affective computing for the automatic classification of depression, bipolar disorder, and Parkinson disease [34-38].

Higher-arousal emotions (eg, fear, anger, and joy) generally induce a higher speech intensity, F0, and speech rate, whereas lower-arousal emotions (eg, sadness and boredom) induce a lower speech intensity, F0, and speech rate (Table 1) [33,39-43]. Other features include a harmonics to noise ratio, which was found unrelated to arousal [44], and jitter, which showed a positive correlation with depression [45]. Arousal has been easiest to detect based on voice acoustics [46]. Discrete emotion recognition based on these features in deep neural networks has also been successful [47]. It is not yet clear whether these features could also discriminate between discrete emotions in simple models [48].

**Table 1:** Expected emotion–speech form correlations.

Emotion	F0^a^-mean	F0-SD	F0-range	F0-rise	F0-fall	Loudness mean	Loudness rise	Loudness fall	Jitter^b^	Shimmer^c^	HNR^d^	Speech rate	Pause duration
Valence													
Arousal	(+)^e^	+	+	+	+	+	+	+				(+=)^f^	(−)^g^
Anger	+	+	+	+	+	+	+	+	+=	+=	+	(+−)^h^	−
Anxiety	+	+−	+	+−	+−	+			+	+	−	+−	+−
Sadness or depression	−	−	−	−	−	−	−	−	+		−	−	+
Stress	+	+	+	+	+	+	+	+				+	−
Happiness	+	+	+	+	+	+	+	+	+=	+=	+	+	−

^a^F0: fundamental frequency.

^b^Deviations in individual consecutive fundamental frequency period lengths.

^c^Difference in the peak amplitudes of consecutive fundamental frequency periods.

^d^HNR: harmonics to noise ratio (energy in harmonic components and energy in noise components).

^e^Positive correlation.

^f^Positive or no correlation.

^g^Negative correlation.

^h^Undirected correlation.

#### Writing Content

Higher valence has repeatedly been associated with more positive and less negative emotion words on a within- and between-person level, along with a higher word count in both natural and induced emotion conditions (Table 2) [28,49-51]. Other studies have demonstrated 1-time links between higher valence and more exclamation marks and fewer negations between persons and between higher valence and less sadness-related words within persons [50,51], although the latter 2 have also been found to be unrelated [28,49]. Pennebaker [52] states that people use more first-person plural pronouns when they are happy.

**Table 2:** Expected emotion–speech and writing content correlations.

Emotion	WC^a^	I	We	You	Negate	Posemo^b^	Negemo^c^	Anx^d^	Anger	Sad	Certain^e^	Swear	Exclam^f^
Valence	(+)^g^	(−)^h^								+		+	+
Arousal													
Anger		+	+	+					+				
Anxiety		+	+	+	+	+	+	+			+		
Sadness		+	+		+			+					
Stress		+		+									
Happiness	+	−								+		+	+
Depression		+	+	+	+	+	+	+			+		

^a^WC: word count.

^b^Posemo: positive emotions.

^c^Negemo: negative emotions.

^d^Anx: anxiety.

^e^Certain: absolutist words.

^f^Exclam: exclamation marks.

^g^Positive correlation.

^h^Negative correlation.

Negative emotion, anxiety, and anger words recur as linguistic markers of anger within and between persons [49,51]. Pennebaker [52] adds to that the use of second-person pronouns. Recurrent linguistic markers of trait anxiety include negative emotion, sadness, and anger words [53,54]. The results with explicit anxiety words are mixed, and some isolated findings suggest a relationship with first-person, negation, swear, and certainty words [53,54]. Momentary and trait sadness have been linked to more negative emotion, sadness, and anger words in multiple studies [28,49,51]. In contrast, they were unrelated to sadness words in daily diaries [51]. A positive correlation existed between stress on one side and negative emotion and anger words between and within persons on the other [51,54]. Anxiety words have been related to stress both on a weekly and daily level [51], but this could not be replicated with trait stress [54]. Apart from the explicit emotion categories, several studies have linked depressive symptoms to the use of *I* words [23,55-58]. Other correlations include more negative emotion words, more swear words, and more negations [53,59,60]. More anxiety, sadness, and anger words were found in 1 study but were not significant in all studies [51,54]. In fact, Capecelatro et al [31] found depression to be unrelated to all Linguistic Inquiry and Word Count (LIWC) emotion categories.

#### Writing Form

Initially, studies concerning typing dynamics used external computer keyboards to predict stress and depression, among other emotions [61-65]. More recent studies have tried to use soft keyboards on smartphones for emotion, depression, and bipolar disorder detection [66-69]. It has been easier to distinguish between broad emotion dimensions—valence in 1 study and arousal in another [66,70].

Despite the high predictive accuracies of deep learning models, separate correlations between emotional states and typing dynamics are small (Table 3). They exist between increased arousal and decreased keystroke duration and latency [70]. The dynamics used in depression detection include a shorter key press duration and latency, with a medium reduction in duration for severe depression but a high reduction for mild depression [61]. No correlation was found between depression and the number of backspaces. For emotions, typing speed was the most predictive feature [66].

**Table 3:** Expected emotion–writing form correlations.

Emotion	Number of characters	Typing speed	Average key press duration	Number of entries	Backspaces	Typing duration
Valence	(+)^a^					
Arousal		+	(−)^b^			−
Anger						
Anxiety						
Sadness						
Stress		+	−	−	−	−
Happiness	+					
Depression			−			

^a^Positive correlation.

^b^Negative correlation.

### This Study

Despite this body of research, crucial questions remain. For instance, most research has focused on between-person relationships, whereas few studies have looked at state emotions within persons. Therefore, it is unclear to what extent mobile-sensed language can help predict moment-to-moment changes within individuals. Previous research has typically also examined particular language features in isolation. As a result, we do not know how the different types of language data compare in their predictive value nor to what extent combining them may enhance the prediction of moment-to-moment and trait emotions.

In this study, we will examine the separate and combined predictive value of 4 mobile-sensed language data sources for detecting momentary emotional experience as well as emotional traits and depression. A 2-week ESM study was designed, querying participants to indicate their valence, arousal, anger, anxiety, sadness, stress, and happiness on their smartphones 10 times a day. In addition, a custom-built app recorded data from several sensors. Relevant to this study, the participants were asked to use the provided custom keyboard software as often as possible and to make a voice recording regarding their emotional state at the end of each ESM survey. On the basis of these data, we will examine how self-reported emotional experience is correlated and can be predicted with spoken and written word use, acoustic voice features, and typing dynamics.

This study goes beyond previous work by comparing and combining all four sources of language behavior: speech, writing, content, and form. In addition, this study will examine the prediction of emotion traits as well as moment-to-moment emotional fluctuations in daily life, providing a comprehensive picture of the potential of language-based smartphone-sensing data for emotion detection.

## Methods

### Participants

Participants were recruited through notices on social media groups and notice boards around university buildings. In this notice, people were directed to a web survey for selection purposes. This web survey queried an email address, age, gender, and questions regarding the inclusion criteria. These entailed Dutch as mother tongue, availability for the duration of the study, ownership of an Android smartphone that supported the sensing app (not iPhone, Huawei, Wiko, Medion, or Xiaomi), always carrying that smartphone, and activating it at least 10 times a day. A total of 230 people completed the web survey, of whom 116 (50.4%) were excluded based on the aforementioned criteria. Of the remaining 114 people, 69 (60.5%) agreed to participate in the study. In the laboratory, 3% (2/69) of participants refused to sign the informed consent, and the installation of the apps failed with another 3% (2/69) of participants, leaving 65 actual participants. For the analyses, an extra inclusion criterion of having answered at least 30 surveys led to the exclusion of another 8% (5/65) of participants. Of the remaining 60 participants, 17 (28%) were men, and 43 (72%) were women (mean age 21.85 years, SD 2.31 years; range 17-32 years).

The participants were reimbursed depending on their cooperation in the study. A maximum of €50 (US $56) could be earned. A total of €10 (US $11.2) were earned after completing some baseline trait questionnaires at the start of the study. Another €5 (US $5.6) could be earned per 10% completed ESM surveys, ending at 80% completed surveys. This is a standard practice in ESM research. This study was approved by the Societal Ethical Commission of Katholieke Universiteit Leuven (G-2018 01 1095).

### Materials

#### Mobile Sensing

A total of 2 apps were installed on each smartphone. The first one, a custom-built app called Actitrack, recorded data from multiple mobile sensors, such as screen locks, light sensors, and location. The software also provided a custom onscreen keyboard display that could be used instead of the default soft keyboard on the host smartphone. This way, the app could register all typing activity with the custom keyboard as it had no access to the default keyboard. Because of the precariousness of these data, privacy measures were taken. All data were securely sent over https to a central server of Katholieke Universiteit Leuven and stored in 2 different files.

This study solely focused on the sensed keyboard and voice data. The participants were asked to use the custom-made keyboard as often as possible to render enough writing data. While doing so, the following variables were stored: content of the message, number of backspaces, number of characters, typing speed, typing duration, average duration of a key press, number of positive emojis, and number of negative emojis.

After each ESM survey, the participants were redirected to the sensing app to record a voice message. In the app, there was a button to start and a button to decline, and the instruction read “Make a recording of about one minute about what you have done and how it made you feel. Good luck!” This meant that keyboard activity was passively sensed the entire time of the study, whereas voice recordings were actively prompted and initiated by the participants. As the keyboard messages and voice recordings might contain sensitive personal information, the files were encrypted separately and could only be stored and handled on computers with an encrypted hard drive.

#### ESM Approach

The second app, MobileQ, delivered the ESM surveys [71]. A total of 10 times a day for 2 weeks, the participants were prompted to answer some questions, including current levels of valence, arousal, anger, anxiety, sadness, stress, and happiness, using a visual analogue scale (0-100). The first notification of each day was sent randomly between 10 AM and 11 AM, including a question about sleep quality. The other 9 surveys were semirandom, dividing the time between 11 AM and 10 PM into 9 equal blocks and randomly programming a beep in each block. Other questions concerned where and with whom the participant was, what they were doing, if the app had worked without problems, and whether something positive or negative had happened since the last survey, but these questions are not analyzed in this paper.

#### Mental Health Survey

At the beginning of the study, each participant completed a mental health and personality survey. In this study, only the depression subscale of the Depression, Anxiety, and Stress Scale (DASS) was used [72]. The DASS contains 21 statements, and the participants must indicate how much these applied to them on a scale of 0 to 3. The depression subscale is an average score of 7 items.

### Procedure

After meeting the inclusion criteria, the participants attended a session in the laboratory. During each session, an informed consent was first proposed and signed. Next, the 2 apps were installed on the participants’ smartphones, and they received a booklet with user instructions and a unique participant number. The booklet included instructions to keep the phone turned on, charge it at night, not lend it to a friend, switch off the screen lock, and be connected to Wi-Fi as much as possible. It also included a guide on how to install and uninstall the apps. Finally, the participants were asked to complete the trait questionnaires. For each participant, the 2-week study began the day after the session, and the apps were automatically deactivated after 15 days. There was an optional feedback session at the end where the participants could receive a debriefing and help with uninstallation. The 60 participants that reached the cutoff of 30 completed surveys responded on average to 109.3 (SD 22) of the 140 notifications, yielding a compliance rate of 78% (mean compliance 0.78, SD 0.16; range 0.26-0.99).

### Data Preprocessing

The voice samples were converted to text files to be able to analyze the words used in speech. The voice recordings were initially transcribed using the open-source transcriber software Kaldi (NVIDIA) [73]; however, as the transcripts contained many language errors, all of them were corrected by hand. These text files were then used for the automated word counting. All following data processing and analyses were performed using R (version 4.0.3; R Foundation for Statistical Computing) [74]. First, all voice recordings and keyboard activities were linked to their corresponding ESM surveys based on their timestamps. If the timestamps were not an exact match, voice recordings within 5 minutes of an ESM timestamp were linked to that corresponding survey. Keyboard activity was binned into intervals ranging from 30 minutes before to 30 minutes after an ESM survey by pooling all messages and summing the typing dynamics except for typing speed and average key press duration, for which the mean was taken. Second, all participants with <30 responses or without a single voice recording or keyboard activity were removed. This left 51 participants with a total of 1015 voice recordings and 59 participants with a total of 3929 keyboard bins. Finally, all used measures were prepared for the momentary- and trait-level analyses. For the momentary-level analyses, all observations were standardized within participants. For the trait-level analyses, all observations of a given participant were aggregated into 1 single observation to be used in a between-person context along with the DASS score. The momentary level thus reflects emotional states from one moment to another, whereas the trait level represents the average mood of the participant over the duration of the study. Standardization happened only over the observations with an ESM survey as well as keyboard or voice recordings.

### Feature Extraction

#### Speech Content

The content of the voice recordings was analyzed using the LIWC software [75]. LIWC is a language processing tool that allows for the automated counting and labeling of words. LIWC counts and categorizes words going from pronouns to swear words to religion- or death-related words. Each category is then presented as a percentage of counted words on the total number of words. In this study, the automatically generated Dutch translation of the LIWC 2015 dictionary was used [76]. Twelve categories were selected based on the reviewed literature: *word count*, *i*, *we*, *you*, *negate*, *posemo*, *negemo*, *anxiety*, *anger*, *sad*, *certain*, and *swear* (Table 4).

**Table 4:** Descriptive statistics of the speech data.

Item		Value, mean (SD; range)
**Emotions^a^**		
	Valence	56.21 (11.3; 22.57 to 83.42)
	Arousal	44.7 (11.35; 18.41 to 77.21)
	Anger	10.63 (9.08; 1.7 to 52.05)
	Anxiety	12.47 (12.62; 1.35 to 56.31)
	Sadness	13.06 (9.38; 1.84 to 43.1)
	Stress	27.58 (15.15; 5.08 to 74.16)
	Happiness	56.44 (11.32; 21.41 to 80.68)
	Depression	0.42 (0.45; 0 to 2.14)
**Speech content^b^**		
	WC (word count)	60.72 (31.76; 4 to 125.63)
	I (first-person singular)	9.44 (3.69; 0 to 19.09)
	We (first-person plural)	0.58 (0.83; 0 to 3.7)
	You (second-person singular)	0.06 (0.11; 0 to 0.41)
	Negate (negations)	1.29 (0.75; 0 to 3.28)
	Posemo (positive emotion words)	3.54 (2.04; 0 to 12.5)
	Negemo (negative emotion words)	0.98 (0.72; 0 to 2.73)
	Anx (anxiety-related words)	0.36 (0.52; 0 to 2.38)
	Anger (anger-related words)	0.27 (0.35; 0 to 1.47)
	Sad (sadness-related words)	0.16 (0.18; 0 to 0.76)
	Certain (absolutist words)	1.59 (1.36; 0 to 7.71)
	Swear (swear words)	0 (0.03; 0 to 0.19)
**Speech form^c^**		
	F0^d^ mean	29.93 (4.26; 20.25 to 40.63)
	F0 SD	0.22 (0.05; 0.13 to 0.42)
	F0 range	7.52 (3.63; 2.29 to 19.4)
	F0 mean rising slope	303.85 (76.4; 126.97 to 556.56)
	F0 mean falling slope	155.13 (50.45; 88.93 to 336.52)
	Loudness mean	0.77 (0.37; 0.19 to 2.1)
	Loudness mean rising slope	12.85 (5.01; 3.43 to 26.76)
	Loudness mean falling slope	10.02 (4.08; 2.52 to 17.81)
	Jitter mean	0.05 (0.01; 0.03 to 0.07)
	Shimmer mean	1.29 (0.16; 1.02 to 1.75)
	HNR^e^ mean	4.61 (2.44; −4.16 to 8.6)
	Voiced segments per second (speech rate)	2.12 (0.48; 0.55 to 3.38)
	Mean unvoiced segment length (pause duration)	0.29 (0.56; 0.11 to 4.16)

^a^Emotions were rated on a visual analogue scale of 0-100, and depression was rated on a scale of 0-3.

^b^Except for word count, all Linguistic Inquiry and Word Count dimensions display percentages of the total word count.

^c^Fundamental frequency measures are logarithmic transformations on a semitone frequency scale starting at 27.5 Hz. Loudness measures are the perceived signal intensity. The harmonics to noise ratio displays an energy-related harmonics to noise ratio and is indicative of voice quality along with jitter and shimmer.

^d^F0: fundamental frequency.

^e^HNR: harmonics to noise ratio.

#### Speech Form

The acoustic features of the voice recordings were extracted using the openSMILE software (audEERING GmbH) [77]. OpenSMILE is an open-source audio feature extraction toolkit with SMILE, which stands for speech and music interpretation by large-space extraction. The newest version, openSMILE 3.0, provides a simpler package for Python. We chose the Geneva Minimalistic Acoustic Parameter Set, which provides some basic statistics such as the mean and SD for a minor set of acoustic features [78]. Thirteen parameters were selected based on the reviewed literature: F0 mean, F0 range, F0 SD, F0 mean rising slope, F0 mean falling slope, loudness mean, loudness mean rising slope, loudness mean falling slope, mean jitter, mean shimmer, mean harmonics to noise ratio, voiced segments per second, and mean unvoiced segment length (Table 4). The first 5 relate to the pitch of the voice, the next 3 concern the loudness, the next 3 define the voice quality or timbre, and the last 2 can be interpreted as speech rate and mean pause duration.

#### Writing Content

The content of the writing was analyzed in the same way as the content of the voice recordings—by using the LIWC software and the 12 chosen categories, adding also *exclamation marks* (Table 5).

**Table 5:** Descriptive statistics of the writing data.

Item			Value, mean (SD; range)
**Emotions^a^**			
	Valence	56.07 (10.88; 22.57-83.42)	
	Arousal	44.34 (11.67; 9.27-77.21)	
	Anger	10.49 (8.8; 1.5-52.05)	
	Anxiety	12.14 (12.2; 0.15-56.31)	
	Sadness	12.82 (9.35; 1.84-43.1)	
	Stress	26.85 (15.12; 3.31-74.16)	
	Happiness	56.31 (10.97; 21.41-80.68)	
	Depression	0.45 (0.48; 0-2.14)	
**Writing content^b^**			
	Positive emojis	1.4 (5.94; 0-45.09)	
	Negative emojis	0.15 (0.26; 0-1.35)	
	WC (word count)	82.4 (58.45; 1.8-358.93)	
	I (first-person singular)	3.21 (1.22; 0-5.31)	
	We (first-person plural)	0.57 (0.34; 0-1.38)	
	You (second-person singular)	2.21 (0.82; 0.52-5)	
	Negate (negations)	1.44 (0.81; 0-3.83)	
	Posemo (positive emotion words)	0.1 (0.11; 0-0.38)	
	Negemo (negative emotion words)	3.48 (1.57; 0-8.25)	
	Anx (anxiety-related words)	0.85 (0.39; 0-1.68)	
	Anger (anger-related words)	0.12 (0.12; 0-0.55)	
	Sad (sadness-related words)	0.26 (0.2; 0-0.81)	
	Certain (absolutist words)	0.24 (0.15; 0-0.65)	
	Swear (swear words)	2.31 (1.06; 0-4.86)	
	Exclam (exclamation marks)	1.56 (1.8; 0-9.26)	
**Writing form^c^**			
	Characters, N	480.11 (293.67; 12.7-1764.5)	
	Typing speed (characters per second)	2.1 (0.55; 1.18-4.68)	
	Average key press duration (ms)	79.95 (16.48; 20.68-122.83)	
	Entries, N	15.37 (10.69; 1-71.92)	
	Total backspaces, N	0.17 (0.06; 0-0.3)	
	Total typing duration (seconds)	2.68 (2.18; 0.44-9.85)	

^a^Emotions were rated on a visual analogue scale of 0-100, and depression was rated on a scale of 0-3.

^b^Except for word count, all Linguistic Inquiry and Word Count dimensions display percentages of the total word count.

^c^Number of backspaces and typing duration are divided by the total number of keystrokes (characters + backspaces).

#### Writing Form

The typing dynamics were immediately recorded during typing without any additional software. The variables extracted from the custom-made keyboard were the number of backspaces, typing duration, typing speed, number of characters, and average duration of a key press (Table 5). The absolute number of backspaces and typing duration were transformed into the relative number on the total number of keystrokes for that bin (characters + backspaces). After binning, the number of keyboard entries (eg, separate messages and notes) collected in that bin was also counted.

### Correlation Analyses

After standardization, pairwise correlations were computed between the emotions on one side and the language features on the other. At the momentary level, this was done by extracting the slopes of multilevel simple linear regressions using the lme4 and lmerTest packages in R with the restricted maximum likelihood modeling. At the trait level, Spearman correlations were applied to the aggregated data set. On each correlation table, a false discovery rate (FDR) correction was applied according to the step-down method by Holm [79].

### Predictive Modeling

Next, we were interested in how well the language features would predict emotional states and traits. The total data set for voice and keyboard separately was divided into an 80% training and 20% test set. We used all the significant correlations of the previous analyses for the 4 language types separately as possible predictors for a given emotion in a linear regression model with a random intercept and varying slopes for participants at the momentary level, allowing predictors to have different values for each participant. When the correlation analysis yielded no significant correlations for an emotion, the 3 most highly correlated features were chosen as possible predictors. A 10-fold cross-validation on the training set was applied to determine which of the possible predictors had an average *P* value of <.05, and those were kept in the model. When there were no predictors with an average *P* value of <.05, the 2 best predictors were chosen to prevent overfitting of the training set. Finally, a model with the chosen predictors was fitted on the total training set, and then we calculated the predictive *R*^2^ based on that model and the test set. The predictive *R*^2^ is calculated as the mean squared error divided by the variance of the data, making it scale-independent:

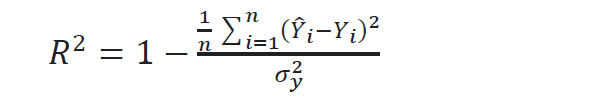



As we noticed that a different split of the test and training sets yielded different results, especially for the trait level, we chose to perform a 50-fold variation of the training and test sets in a bootstrap-like manner. This means we randomly created 50 different splits of the observations into 80% training and 20% test sets.

## Results

### Descriptives

#### Speech

A total of 51 participants (51/60, 85%) recorded between 1 and 96 voice samples on the total number of ESM surveys they completed, with an average compliance rate of 19% (speech mean 0.19, SD 0.21; range 0.01-0.94). Within participants, there was a significant correlation between the day of the study (1 to 14) and the number of voice recordings (*r*=−0.36; *P*<.001), meaning that compliance decreased during the study. For the descriptive statistics of all speech measures, we looked at the distribution of the within-person averages (Table 4). The participants showed sufficient variability in their emotions. *I* and *posemo* were the most counted words, although, in general, the LIWC dimensions only accounted for a small share of the total amount of spoken words. When looking at depression, we saw a large cluster of DASS depression scores between 0 and 0.75 and then 6 sparse points reaching >0.75. The maximum of the scale was 3, which could mean that our sample lacked the sensitivity to register any significant relationships between depressive symptoms and the 4 language types.

#### Writing

A total of 59 participants (59/60, 98%) used the custom-made keyboard between 5 and 117 times in the hour around their completed ESM surveys, with an average use rate of 60% (writing mean 0.60, SD 0.21; range 0.07-0.95). Here, again, use declined throughout the study within participants (*r*=−0.23; *P<*.001). Similar to the speech data, for the descriptive statistics, we looked at the distribution of the within-person averages (Table 5). Overall, this sample showed the same depression and emotion distributions as the speech sample. *I*, *negemo,* and *swear* were the most counted words, although, again, the LIWC dimensions in general only accounted for a small share of the total amount of written words.

### Correlation Analyses

#### Speech Content

**Figure 1:**
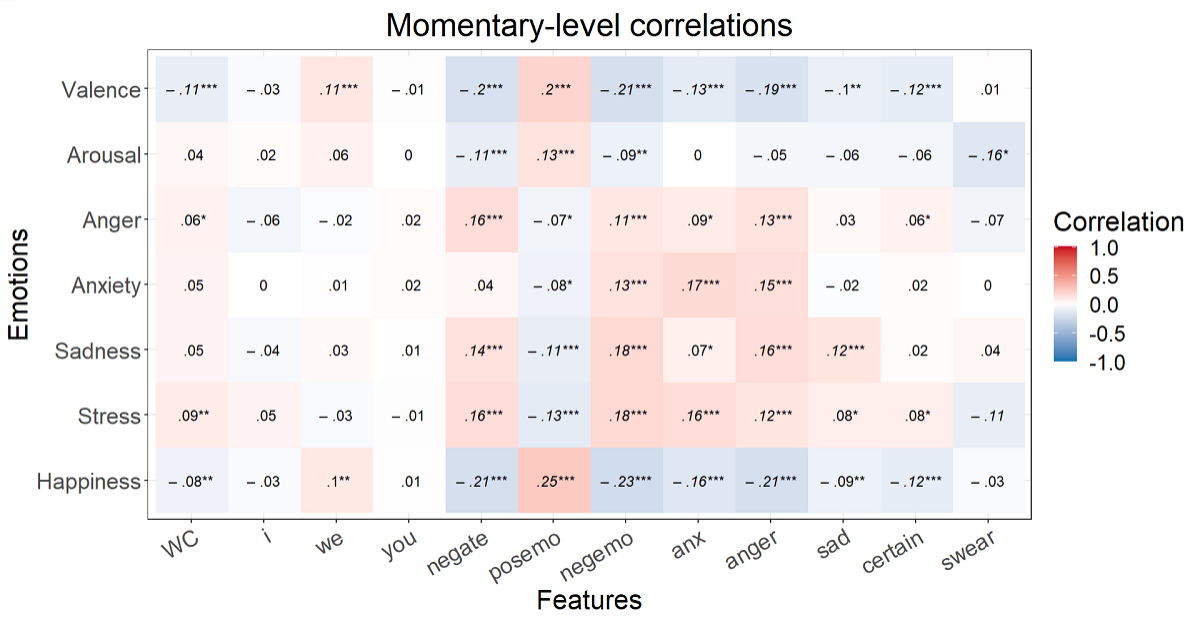
Multilevel correlations between the state emotions and speech content variables (n=1015). *P<.05, **P<.01, ***P<.001. Italicized values are significant after false discovery rate correction. Anger: anger-related words; anx: anxiety-related words; certain: absolutist words; I: first-person singular; negate: negations; negemo: negative emotion words; posemo: positive emotion words; sad: sadness-related words; swear: swear words; WC: word count; we: first-person plural; you: second-person singular.

**Figure 2:**
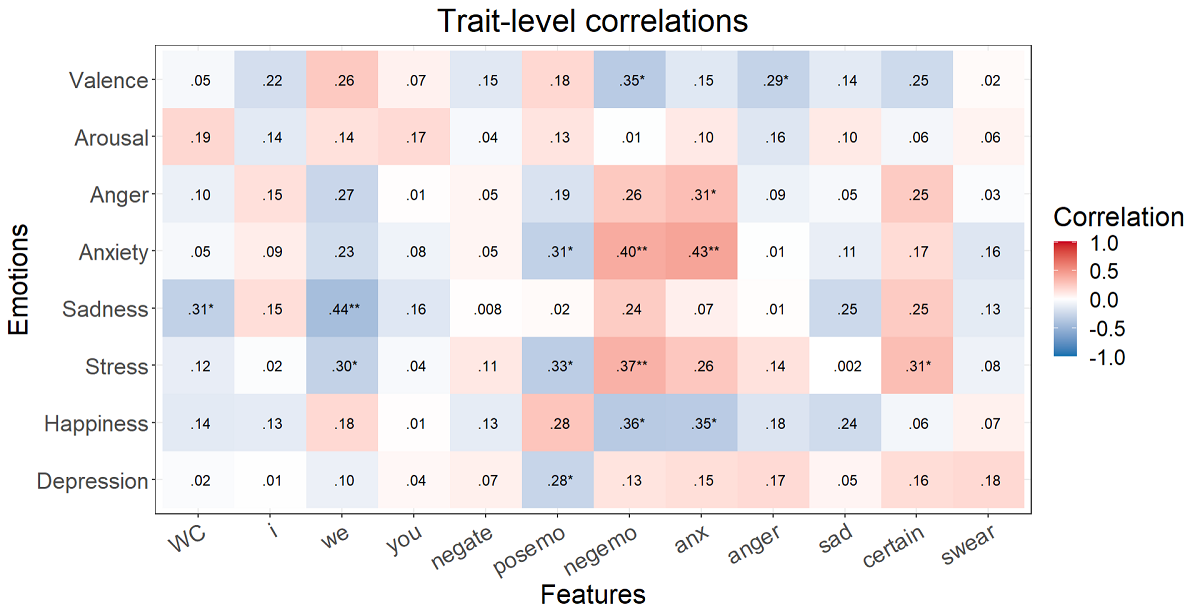
Spearman correlations between the trait emotions and speech content variables (n=51). *P<.05, **P<.01, ***P<.001. Italicized values are significant after false discovery rate correction. Anger: anger-related words; anx: anxiety-related words; certain: absolutist words; I: first-person singular; negate: negations; negemo: negative emotion words; posemo: positive emotion words; sad: sadness-related words; swear: swear words; WC: word count; we: first-person plural; you: second-person singular.

After the FDR correction at the momentary level, *P*<.001 for all significant correlations mentioned here. Higher valence correlated with a lower word count; more *we* and positive emotion words; and fewer negations and negative emotion, anxiety, anger, and certainty words (Figure 1). Happiness showed the same relationships without word count and *we*. Arousal was only correlated with fewer negations and more positive emotion words. Anger showed positive correlations with negations, negative emotion words, and anger words. Anxiety was positively correlated with negative emotion, anger, and anxiety words. More sadness was associated with more negations and negative emotion, anger, and sadness words and with fewer positive emotion words. Finally, stress displayed the same correlations as sadness with anxiety instead of sadness words. At the trait level, some higher correlations arose at first but, after the FDR correction, no correlation was significant (Figure 2).

#### Speech Form

**Figure 3:**
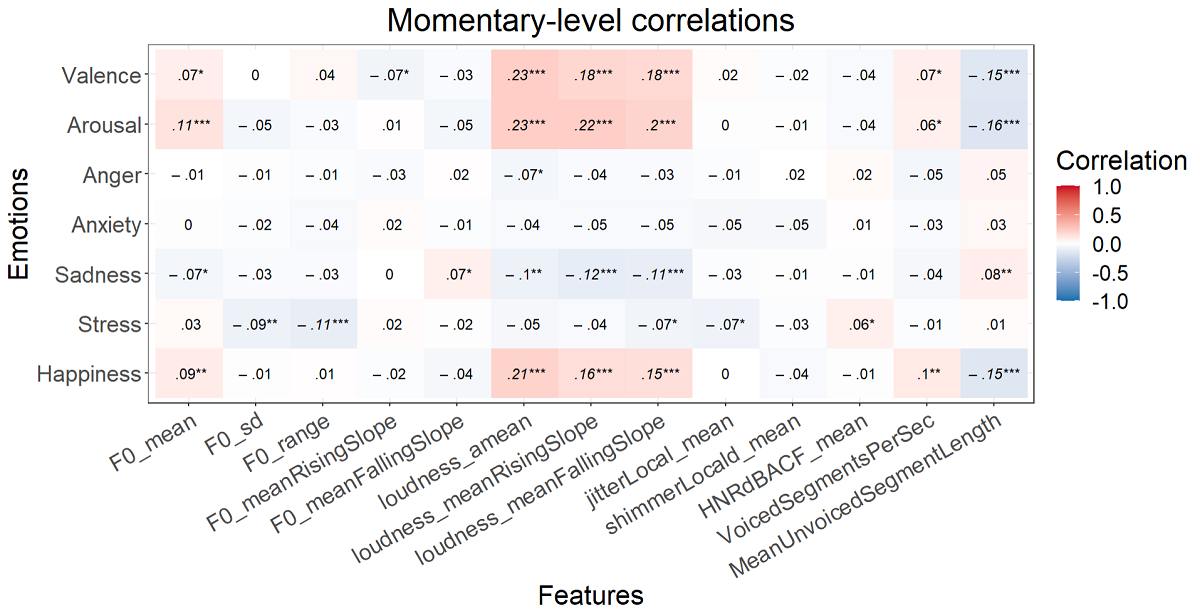
Multilevel correlations between the state emotions and speech form variables (n=1015). *P<.05, **P<.01, ***P<.001. Italicized values are significant after false discovery rate correction.

**Figure 4:**
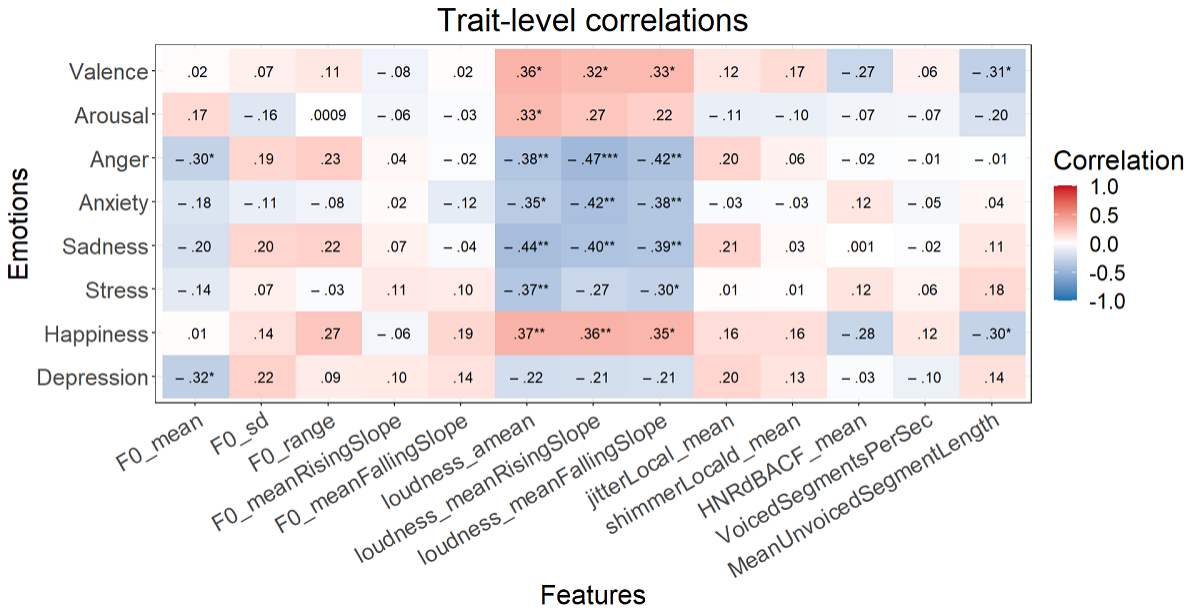
Spearman correlations between the trait emotions and speech form variables (n=51). *P<.05, **P<.01, ***P<.001. Italicized values are significant after false discovery rate correction.

After the FDR correction at the momentary level, *P*<.001 for all significant correlations mentioned here. Higher valence correlated with a higher mean loudness, mean loudness rising slope, and mean loudness falling slope, and a lower mean unvoiced segment length (Figure 3). Happiness showed the same relationships. Arousal correlated with higher values of all 3 loudness measures and a lower mean unvoiced segment length. Anger and anxiety showed no significant correlations after FDR correction. More sadness was associated with a lower mean loudness rising slope and mean loudness falling slope. Finally, stress displayed a significant correlation with a lower F0 range. At the trait level, the correlation values again increased, but none of these were significant (Figure 4).

#### Writing Content

**Figure 5:**
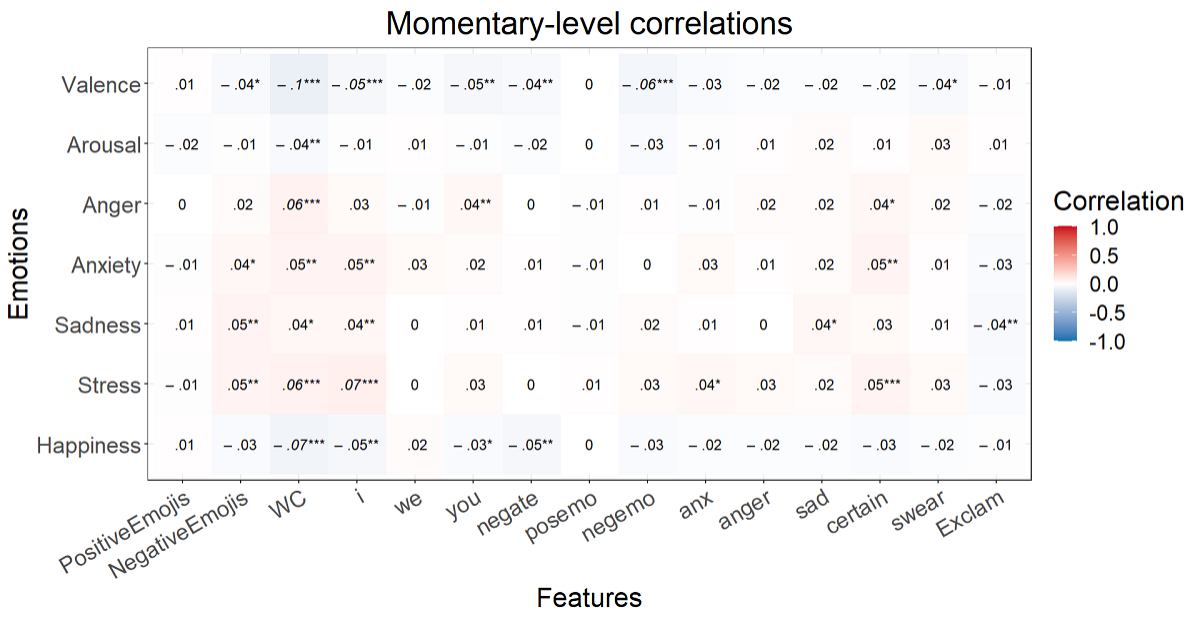
Multilevel correlations between the state emotions and writing content variables (n=3929). *P<.05, **P<.01, ***P<.001. Italicized values are significant after false discovery rate correction. Anger: anger-related words; anx: anxiety-related words; certain: absolutist words; exclam: exclamation marks; I: first-person singular; negate: negations; negemo: negative emotion words; posemo: positive emotion words; sad: sadness-related words; swear: swear words; WC: word count; we: first-person plural; you: second-person singular.

**Figure 6:**
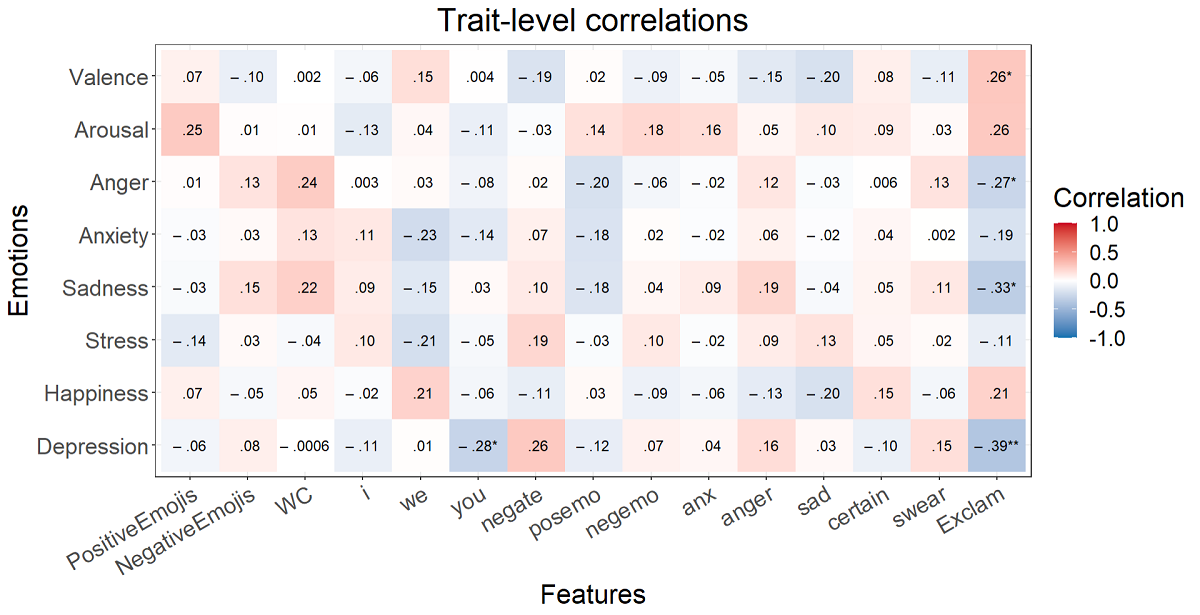
Spearman correlations between the trait emotions and writing content variables (n=59). *P<.05, **P<.01, ***P<.001. Italicized values are significant after false discovery rate correction. Anger: anger-related words; anx: anxiety-related words; certain: absolutist words; exclam: exclamation marks; I: first-person singular; negate: negations; negemo: negative emotion words; posemo: positive emotion words; sad: sadness-related words; swear: swear words; WC: word count; we: first-person plural; you: second-person singular.

After the FDR correction at the momentary level, *P*<.001 for all significant correlations mentioned here. Higher valence correlated with a lower word count and less first-person singular use (Figure 5). Happiness only correlated with a lower word count. Arousal, anxiety, and sadness showed no significant correlations after FDR correction. More anger was associated with a higher word count. Finally, stress displayed a correlation with a higher word count and first-person singular use. At the trait level, none of the correlations were significant (Figure 6).

#### Writing Form

**Figure 7:**
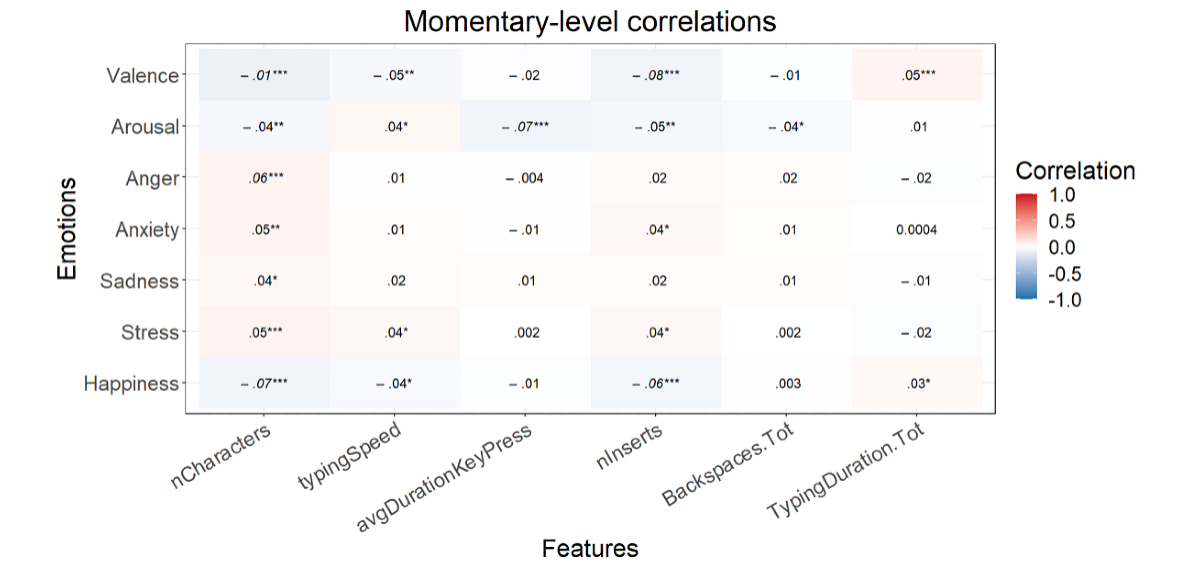
Multilevel correlations between the state emotions and writing form variables (n=3929). *P<.05, **P<.01, ***P<.001. Italicized values are significant after false discovery rate correction. AvgDurationKeyPress: average key press duration; Backspaces.Tot: backspaces divided by the total amount of keystrokes (characters + backspaces); nCharacters: number of characters; nEntries: number of entries; TypingDuration.Tot: typing duration divided by the total amount of keystrokes (characters + backspaces).

**Figure 8:**
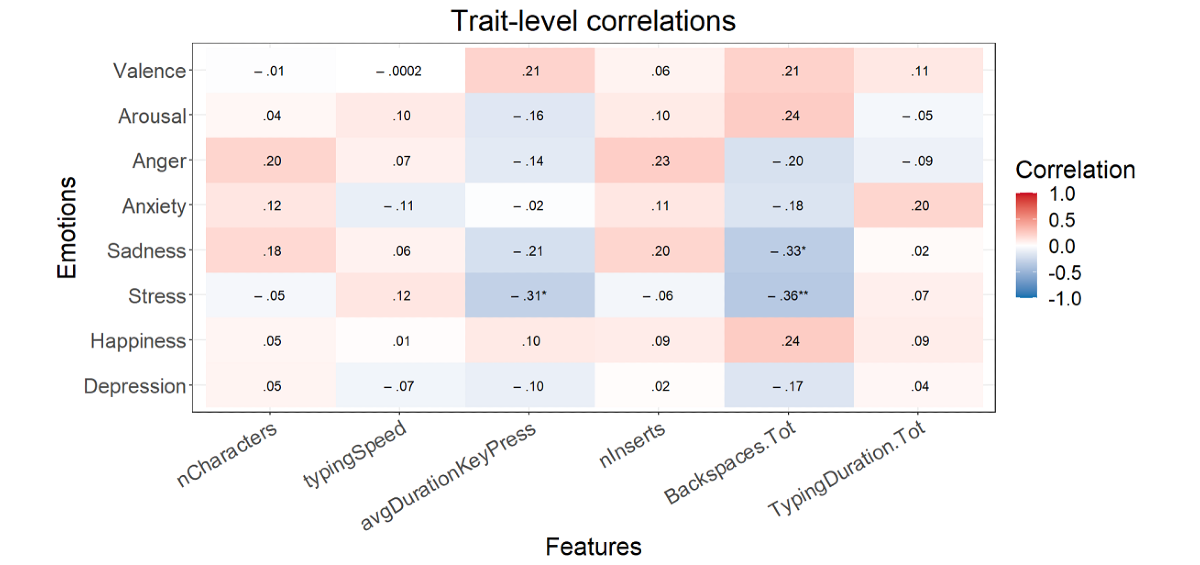
Correlations between the trait emotions and writing form variables (n=59). *P<.05, **P<.01, ***P<.001. Italicized values are significant after false discovery rate correction. AvgDurationKeyPress: average key press duration; Backspaces.Tot: backspaces divided by the total amount of keystrokes (characters + backspaces); nCharacters: number of characters; nEntries: number of entries; TypingDuration.Tot: typing duration divided by the total amount of keystrokes (characters + backspaces).

After the FDR correction at the momentary level, *P*<.001 for all significant correlations mentioned here. Higher valence and happiness correlated with a lower number of characters and keyboard entries (Figure 7). Arousal displayed a correlation with a shorter average key press duration. Anger correlated with a higher number of characters. Anxiety, sadness, and stress showed no significant correlations. At the trait level, no correlations were significant after FDR correction (Figure 8).

### Predictive Modeling

**Figure 9:**
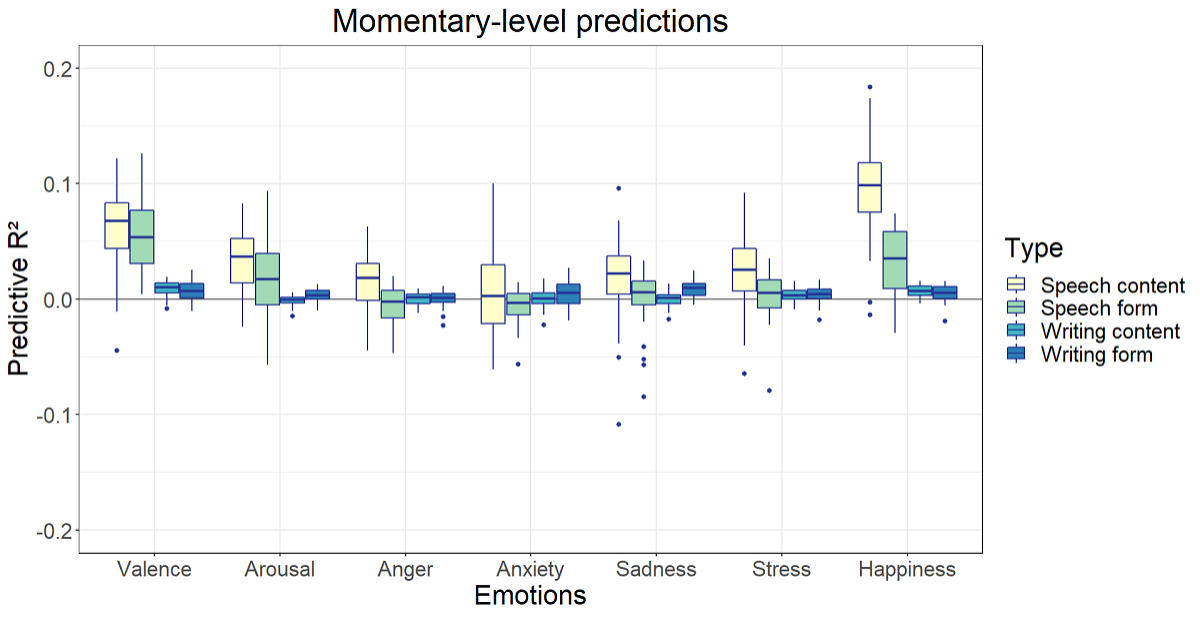
Predictive *R*^2^ of language data at the momentary level.

**Figure 10:**
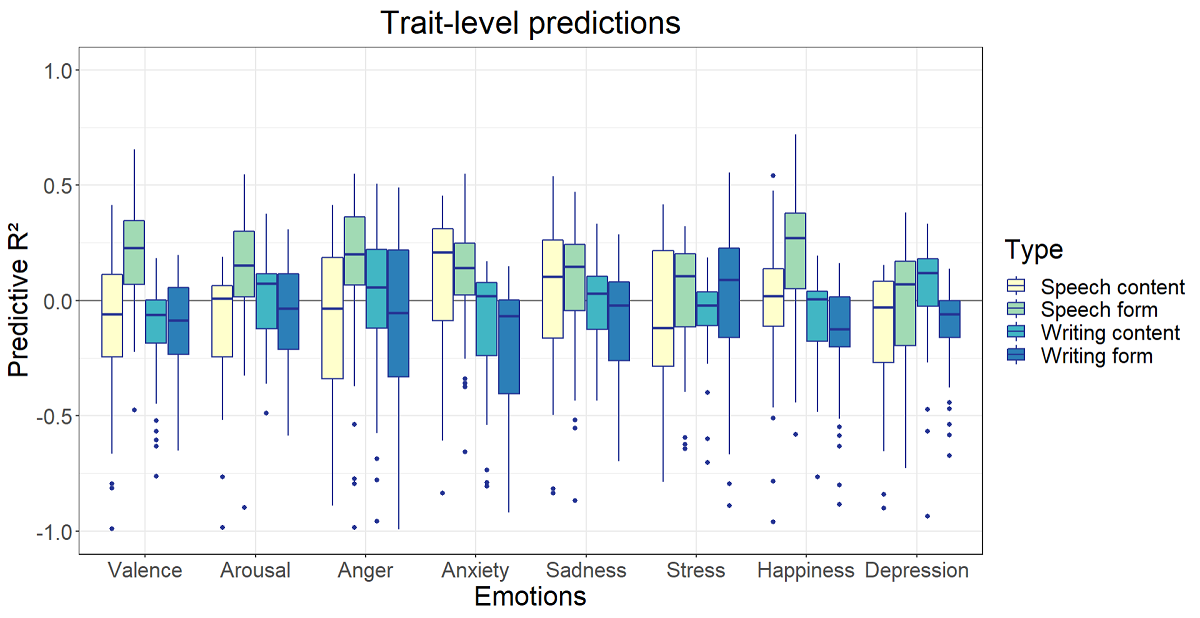
Predictive *R*^2^ of language data at the trait level.

The highest predictive *R*^2^ at the momentary level was found for the prediction of happiness based on speech content (*R*^2^ mean 0.10, SD 0.04; Figure 9) followed by the prediction of valence based on speech content (*R*^2^ mean 0.06, SD 0.03) and speech form (*R*^2^ mean 0.05, SD 0.03). The mean *R*^2^ values of speech content models varied between 0.01 and 0.10, those of speech form varied between −0.01 and 0.05, those of writing content varied between 0 and 0.01, and those of writing form varied between −0.0002 and 0.01. At the trait level, the speech form models performed best, with the highest predictive *R*^2^ for the predictions of valence (*R*^2^ mean 0.16, SD 0.30), happiness (*R*^2^ mean 0.14, SD 0.40), and arousal (*R*^2^ mean 0.13, SD 0.25). All other mean predictive *R*^2^ values were negative except for the speech form prediction of stress (*R*^2^ mean 0.02, SD 0.25) and the speech content prediction of valence (*R*^2^ mean 0.01, SD 0.39; Figure 10).

**Figure 11:**
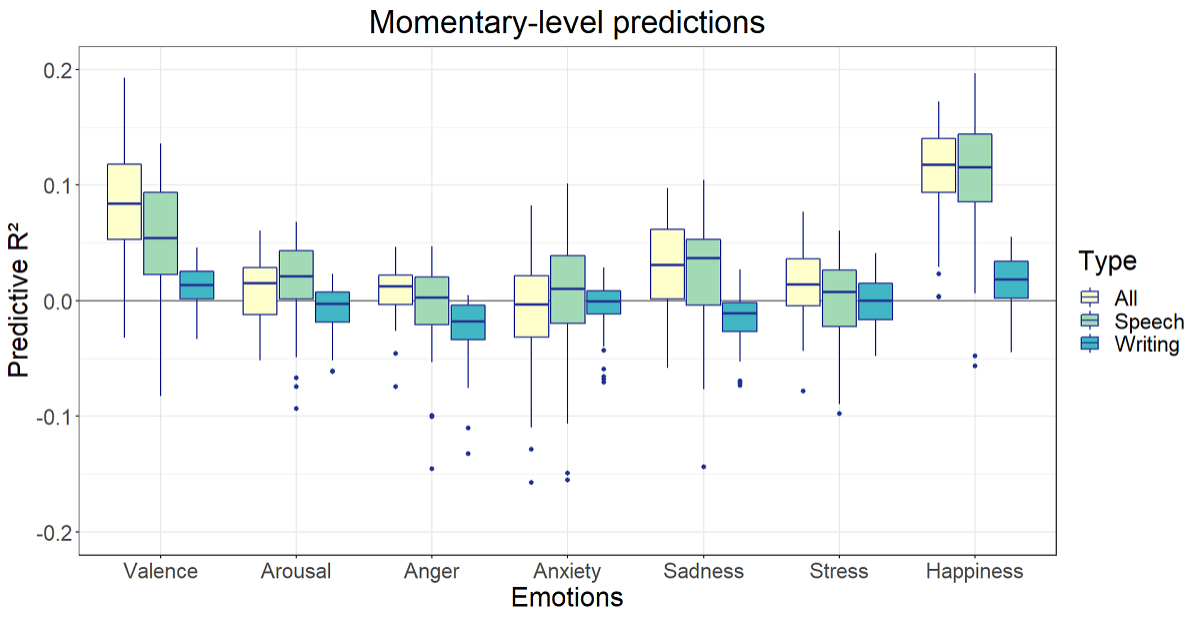
Predictive *R*^2^ of combined language data at the momentary level.

**Figure 12:**
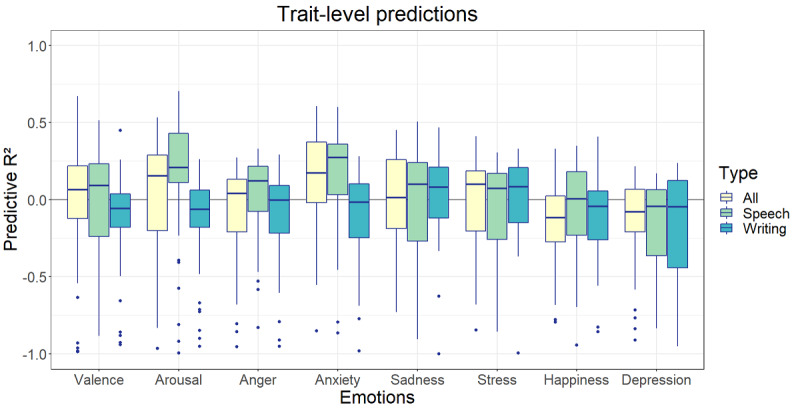
Predictive *R*^2^ of combined language data at the trait level.

Afterward, this process was repeated for the speech content and form features combined in 1 model, the writing content and form features combined in 1 model, and all 4 language features combined in 1 model (Figures 11 and 12). The highest predictive *R*^2^ at the momentary level was found for the prediction of happiness based on all language features (*R*^2^ mean 0.11, SD 0.04) followed by the prediction of happiness based on speech features (*R*^2^ mean 0.11, SD 0.06) and the prediction of valence based on all language features (*R*^2^ mean 0.09, SD 0.05). The mean predictive *R*^2^ values of speech models varied between −0.01 and 0.11, those of writing models varied between −0.02 and 0.02, and those of all features varied between −0.02 and 0.11. At the trait level, the speech models performed best, although only two of the mean predictive *R*^2^ values were >0: the speech prediction of arousal (*R*^2^ mean 0.08, SD 0.50) and the altogether prediction of arousal (*R*^2^ mean 0.03, SD 0.49).

## Discussion

In this study, we investigated the potential of mobile-sensed language features as unobtrusive emotion markers. We looked at pairwise multilevel correlations between emotions or mood and language features—distinguishing between speech content, speech form, writing content, and writing form—and at the (combined) predictive performance of those features in regression models.

### Correlation Analyses and Predictive Modeling

#### Speech Content

Most of the significant correlations were found between speech content features and momentary emotions but were rather low, varying between |0.11| and |0.25|. However, they are in the range of those found in previous studies [30,32]. Most of these significant correlations were found for state valence and happiness, which is also in line with the literature. We found that the explicit emotion LIWC dimensions had the strongest correlations but did not find evidence of a relationship between pronoun use and emotion [52]. We expected to find at least some correlations with pronouns or negative emotion words at the trait level [28,29,31,32], but no correlations were significant after FDR correction.

#### Speech Form

Speech form and momentary emotions also displayed some significant correlations, ranging from |0.11| to |0.23|. Most of the literature has focused on discriminating high-arousal from low-arousal emotions [33,39-41]. In this study, arousal was indeed represented, but so were valence and happiness. However, anger was not. We expected F0, loudness, and speech rate to be important; however, in this study, only the loudness measures and pause duration were notable. At the trait level, nothing was significant. This is surprising given that most of the literature on speech form is based on between-person research.

#### Writing Content

Writing content features showed only a few weak significant correlations. Varying between |0.05| and |0.10|, these were lower than expected yet not entirely surprising given the mixed results throughout previous work [28,51]. Valence was again the best represented; however, in contrast to speech content, the first-person singular was most notable in writing along with word count. At the trait level, the exclamation marks seemed promising at first but turned nonsignificant after FDR correction, meaning this study was not able to replicate earlier findings with anxiety and depression [23,53,55-60].

#### Writing Form

Writing form showed the least amount of significant correlations, also in the range of |0.05| to |0.10|. In the literature, typing speed and average key press duration have been seemingly linked to emotions; however, in this study, the number of characters and the number of keyboard entries were the most telling. They followed the direction of the word count correlations. Here, again, valence and happiness showed the most correlations, and the complete trait level was nonsignificant.

#### Predictive Modeling

As could be expected based on the number of significant correlations, valence and happiness showed the highest predictive *R*^2^ values at the momentary level. In addition, the speech content models performed best followed by the speech form models. The predictive *R*^2^ estimations of the writing content and form models always stayed close to 0, although their variation was smaller (Figure 9). This is all in line with the previously found correlations. In addition, the size of the values followed the trend of the correlations and remained rather low—at most, 10% of peoples’ state emotions can be predicted based on their momentary language.

When combining multiple types of momentary language data into the same models, writing does not contribute to better predictions. Combining speech content and form features yields more or less the same results as their separate models, whereas adding writing content and form features does not further improve the predictive performance. An important remark here is that not all ESM surveys with voice recordings had additional keyboard activity. Because of this, the data set was further reduced in size, which might contribute to the fact that the combined models seemingly have no added value.

No significant correlations were found at the trait level. In addition, by aggregating, the number of data points was reduced from multiple observations to a single observation per participant. As a result, our expectations for trait predictive performance were lower than those for the momentary models. As can be seen in Figures 10 and 12, the estimations of the predictive *R*^2^ based on varying training and test sets show a larger variation than those of the momentary models. Moreover, they are clustered around 0 with numerous negative outliers, indicating regular overfitting of the training set. There was one type of data that performed better than the others: >75% of the predictive *R*^2^ estimations based on the speech form models for valence, arousal, anxiety, and happiness performed >0, indicating at least some predictive value.

Overall, the found relationships were largely in the predicted direction but were very modest in size. For speech, these values are more or less in line with previously obtained results; however, writing performed below expectations. There are 3 main differences between voice recordings and keyboard activity that might account for this. One is the nature of collection—voice recordings were deliberately voiced, whereas keyboard activity was unobtrusively recorded. Second, keyboard activity was gathered without any instruction, whereas voice recordings came with the explicit instruction for the participants to say what they were doing and how they felt. Finally, although LIWC was able to categorize on average 87.17% (SD 38.83%) of the spoken words, it only recognized on average 54.23% (SD 25.81%) of the text messages because of typos and other distortions.

A second dichotomy exists between the momentary and trait values. Previous work has often focused on between-group designs; however, this study could only record significant within-person correlations. At the trait level, we found no significant correlations, and predictive trait models showed more variability and 0 or negative predictive *R*^2^ values. Possibly, by aggregating the emotion and language data, important context data of their relationship were lost, and moment-to-moment tendencies were flattened out. For predictive modeling, trait level also meant a reduction in data points to train and test a model. The repeated redistribution of a small number of participants over the training and test sets will induce larger changes than a larger data set. Furthermore, momentary-level models are trained and tested within persons, whereas trait-level models are trained and tested between persons. The overfitting of predictive models at the trait level suggests that the participants’ emotions and language use were too dissimilar to be encapsulated in 1 model (except perhaps for speech form).

### Limitations and Future Directions

The first limitation entails that data collection was dependent on the participants’ willingness to use the custom-made keyboard instead of their default one and to make recordings. This reduced the number of observations and created an unbalanced data set. Ideally, the smartphone’s own keyboard and microphone could be activated and logged at will. This is impossible because of technical and ethical constraints. A solution might be to link reimbursement directly to the provision of valid data in the form of keyboard use or voice recordings, although this might lead too much to a perception of coercion.

A second limitation lies in the software used. We worked with LIWC as it is widely used in the literature and provides a fast and easy-to-use interface. A downside is that it only recognizes single words and not phrases. When the participants talked about feeling *not too happy*, LIWC scored this as a positive emotion and a negation. When looking at the correlations, this did not seem to pose a direct problem in this study, although it could add noise and reduce statistical significance. What might be more problematic is the language the participants used in their texting: abbreviations, typos, and neologisms. Although LIWC 2015 has a netspeak dimension, the average word recognition of writing was only 54.23% (SD 25.81%). In future studies, one might consider preprocessing all writing by hand, although this will be a very time-consuming task.

A third limitation is inherent to the sample of participants. Despite the strong representation of depression and language use in the literature, this study was not able to link depressive symptoms to any language feature. Replicating this study in a more diverse or clinical population might yield other results for depression.

Finally, language is strongly dependent on the chosen medium. Talking to a smartphone with a specific instruction restrains the natural flow of language and can compromise the generalizability of these findings. More technically, this also means that the participants would sometimes talk softly in a quiet room, whereas they might be screaming over the noise in another recording. We should keep in mind the fact that loudness is as much a factor of the environment as it is of the voice. Then again, this context might also say something about the emotional experience in itself.

### Conclusions

This study investigated the relationship between self-reported emotions and 4 types of mobile-sensed language. The found correlations and predictive performances were overall weak, remaining <0.25. The best-performing language type was speech content, which displayed the largest number of significant correlations and the largest predictive *R*^2^ values at the momentary level, followed by speech form. At the trait level, no significant correlations were found, resulting in unreliable predictive models. Only speech form models were able to reach a mean predictive *R*^2^ value >0 at the trait level. Among the studied emotions, valence and happiness showed the most significant correlations and predictability. In conclusion, this means that the potential of this particular set of mobile-sensed language features as emotion markers, although promising, remains rather low.

## Abbreviations

DASS: Depression, Anxiety, and Stress Scale
ESM: experience sampling method
F0: fundamental frequency
FDR: false discovery rate
LIWC: Linguistic Inquiry and Word Count
